# Coral geochemical response to uplift in the aftermath of the 2005 Nias–Simeulue earthquake

**DOI:** 10.1038/s41598-024-57833-1

**Published:** 2024-04-15

**Authors:** Sindia M. Sosdian, Michael K. Gagan, Danny H. Natawidjaja, Alena K. Kimbrough, Bambang W. Suwargadi, Hamdi Rifai, Heather Scott-Gagan, Dudi Prayudi, Imam Suprihanto, Wahyoe S. Hantoro

**Affiliations:** 1 School of Earth and Environmental Sciences, Cardiff University, Cardiff, CF10 3AT UK; 2 Research School of Earth Sciences, The Australian National University, Canberra, ACT 2601 Australia; 3 School of Earth, Atmospheric and Life Sciences, University of Wollongong, Wollongong, NSW 2522 Australia; 4 School of the Environment, The University of Queensland, St Lucia, QLD 4072 Australia; 5 Research Center for Geological Disaster, National Research and Innovation Agency (BRIN), Bandung, 40135 Indonesia; 6 Research Center for Geotechnology, Indonesian Institute of Sciences (LIPI), Bandung, 40135 Indonesia; 7 Department of Physics, Universitas Negeri Padang, Padang, 25131 Indonesia

**Keywords:** Biogeochemistry, Ecology, Environmental sciences

## Abstract

On 28 March 2005, the Indonesian islands of Nias and Simeulue experienced a powerful M_w_ 8.6 earthquake and coseismic uplift and subsidence. In areas of coastal uplift (up to ~ 2.8 m), fringing reef coral communities were killed by exposure, while deeper corals that survived were subjected to habitats with altered runoff, sediment and nutrient regimes. Here we present time-series (2000–2009) of Mn/Ca, Y/Ca and Ba/Ca variability in massive *Porites* corals from Nias to assess the environmental impact of a wide range of vertical displacement (+ 2.5 m to − 0.4 m). High-resolution LA-ICP-MS measurements show that skeletal Mn/Ca increased at uplifted sites, regardless of reef type, indicating a post-earthquake increase in suspended sediment delivery. Transient and/or long-term increases in skeletal Y/Ca at all uplift sites support the idea of increased sediment delivery. Coral Mn/Ca and Ba/Ca in lagoonal environments highlight the additional influences of reef bathymetry, wind-driven sediment resuspension, and phytoplankton blooms on coral geochemistry. Together, the results show that the Nias reefs adapted to fundamentally altered hydrographic conditions. We show how centuries of repeated subsidence and uplift during great-earthquake cycles along the Sunda megathrust may have shaped the modern-day predominance of massive scleractinian corals on the West Sumatran reefs.

## Introduction

Tectonic activity along subduction zones produces the most powerful earthquakes observed on Earth. A foremost example of these megathrust earthquakes is the 2004 Great Boxing Day earthquake in Indonesia which devastated infrastructure and human lives in 14 countries. Beyond the impact on society, these events have sudden and lingering impacts such as vertical crustal displacement (i.e., uplift, subsidence) and tsunamis which heavily impact coastal zones and coral reef systems. Within the life-span of coral reefs, these abrupt coseismic disturbances and concomitant environmental changes play a major role in shaping coasts and the speciation and distribution of reef-building scleractinian corals. For example, subduction zone earthquakes can raise shallow coral reef communities above sea level and lead to their demise by aerial exposure with loss in range of suitable habitat^1–6^. Shifts to distinct new reef zones with altered environmental conditions (i.e., light, turbidity) can encourage colonization by filamentous algae and physiological stress^1,7,8^. Coseismic landslides and tsunami-related effects associated with earthquakes increase sediment delivery to coastal reefs and alter biogeochemical conditions^9–14^. Long-term impacts result in recolonization of impacted habitats and shifts in marine foundation species and species community structure^15–18^. Indeed, long-term patterns in tropical Indo-Pacific coral reef biodiversity are tied to tectonic activity^19–21^.

**Figure 1:**
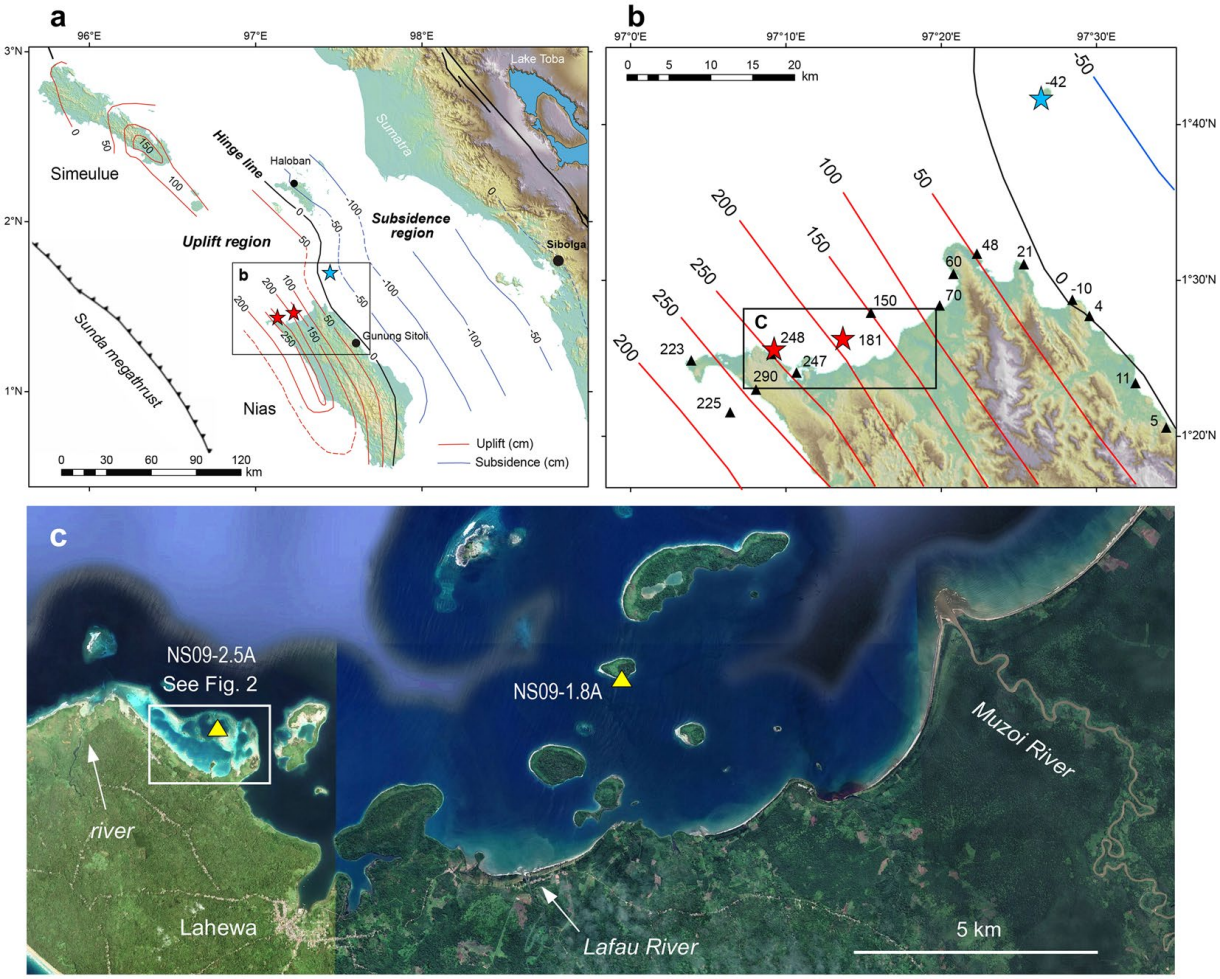
Attributes of the study area. (**a**) Location of Nias, the Sunda megathrust and coseismic uplift and subsidence for the 28 March 2005 Nias-Simeulue earthquake (after Briggs et al.^2^). (**b**) Location of coral drill-sites NS09-2.5A and NS09-1.8A (red stars) and NS09-M0.4A (blue star). Map contours are in centimetres, based on analysis of coral microatoll elevations^2^ (triangles). Site locations include uplift (2.5 m, 1.8 m) and subsidence (− 0.4 m). (**c**) Location of the 2.5A and 1.8A uplift sites relative to riverine sediment sources. 1.8A is in proximity (~ 7 km) to the largest river (Muzoi), and also a smaller river (Lafau). 2.5A is proximal (~ 3 km) to a small unnamed river. Map image is for December 2022 on Google Earth Pro 7.3.6.9345 (2022 version) at http://www.google.com/earth/index.html (accessed August 6, 2023).

The Sunda megathrust, a 5500-km-long subduction zone fault west of Sumatra, Indonesia, produces great-earthquakes about every two centuries^22^ with the potential to shape the coastal environment and coral reef communities across large spatial scales (Fig. 1). Powerful megathrust earthquakes occurred in December 2004 (M_w_ 9.1), and three months later in March 2005 (M_w_ 8.6), centered near the offshore islands of Simeulue and Nias, resulting in significant coseismic uplift (+ 2.8 m) and tsunami damage^2,3^. Uplift during both earthquakes led to extensive reef mortality and corals from deeper habitats colonized the reef flat zone altering the ecological dynamics of fringing reef ecosystems^3^. Vertical displacement altered the coastal geomorphology and distribution and movement of sediment in the nearshore environment, including loss of intertidal habitats minimizing the buffering capacity of this ecosystem^3^.

Despite the potential impact of earthquakes on coral reef systems there is a lack of information on relevant spatial and temporal scales. Instrumental data documenting these instantaneous events and their impacts are limited to continuous global positioning station (GPS) data to document coseismic crustal motion^2^. Also, most environmental assessments to date are visual and provide only a snapshot of coral reef conditions, water quality and health post-earthquake^3,9,12^. Longer-term observations, spanning years, are needed to provide knowledge of environmental change pre- and post-earthquake and potential interaction between land displacement, coastal hydrographic conditions, and coral reef communities.

Long-lived *Porites* spp. corals inhabit tectonically active regions and experience recurring amounts of uplift and subsidence and associated environmental stress over time. *Porites* corals can live for hundreds of years, and those that remain submerged after large earthquakes provide an archive of earthquake-induced environmental change and coral physiological response. For instance, the growth response of *Porites* microatolls has provided a record of interseismic and coseismic crustal motion with a ~ 200 years recurrence cycle along the Sunda megathrust^22–26^. Although the isotopic and elemental compositions of *Porites* skeletons have been shown to track past environmental change^27–29^, their application in earthquake prone regions is limited. Recent work has shown that shifts in skeletal carbon-isotope ratios in *Porites* reflect changes in light exposure following coseismic vertical displacements during the 2005 Nias-Simeulue earthquake^8,30^. Also, skeletal Mg/Ca in *Porites* has been linked to tsunami-induced algal blooms and/or presence of endolithic algae^8^. However, to date, there are no coral records that characterize post-earthquake marine geochemical change in coral reef settings.

Records of Mn, Y and Ba in coral skeletons have the potential to provide information on earthquake related changes in sediment delivery to nearshore areas and associated shifts in sediment dynamics within reef environments. Delivery of sediments via runoff to the coastal zone is the primary source for these elements in nearshore regions, and coral proxy records have been widely used to reconstruct terrigenous runoff on seasonal and longer timescales in the tropics^29^. For instance, Ba/Ca in corals reflects changes in Ba in the surface-ocean reef environment related to a range of processes such as upwelling^31,32^, river discharge^33–35^, wind/wave associated sediment resuspension^36^ and primary productivity and barite formation^37,38^. Mn/Ca in corals has been shown to track Mn cycling associated with large erosive events and delivery of suspended particulate matter through riverine discharge^39–41^. The residence time of Y in the estuarine zone is longer and gradual release and recycling of Y implies coral Y/Ca will record longer-term changes in dissolved Y concentrations related to environmental perturbations^42–47^, such as major terrestrial runoff changes or episodes of large scale erosion.

Here, we present the first records of coral Mn/Ca, Y/Ca and Ba/Ca as evidence of environmental shifts in reef systems in the aftermath of the great Nias-Simeulue earthquake of 28 March 2005. Maximum vertical displacement was centered on the northwest coast of Nias (+ 2.8 m), while a trough of subsidence (to − 1.1 m) occurred between the island and mainland Sumatra^2^ (see Fig. 1b). We explore ~ 9-year-long geochemical records for six massive *Porites* corals positioned along a transect of vertical displacement from + 2.5 m uplift to − 0.4 m subsidence (Fig. 1, Table 1), and across different reef environments (e.g., lagoonal, fringing). We showcase the potential for Mn/Ca, Y/Ca and Ba/Ca to geochemically fingerprint shifts in sediment dynamics and reef environments following the earthquake. The new geochemical records, along with information about great-earthquake recurrence intervals in West Sumatra, allow us to assess the long-term environmental impact of vertical tectonic motion on coral reef expansion and retreat in earthquake prone regions.

**Table 1:** Nias coral core information including environment description and age modelling approach. Age models are based on Gagan et al.^30^ δ^13^C records for site 1.8A and a combination of Ba/Ca and coral core features for sites 2.5A and M0.4A.

Locality	Coral ID	Location	Vertical movement (m)	Environment	Extension rate (mm/y)	Earthquake tie point (mm)	Age model
Lahewa	NS09-2.5A-1	N 1° 25′ 40.9″, E 97° 09′ 18.2″	+ 2.5	Reef lagoon	17	65	Ba/Ca cyclicity
Lahewa	NS09-2.5A-3	N 1° 25′ 41.6″, E 97° 09′ 17.3″	+ 2.5	Reef lagoon	14	63	Ba/Ca cyclicity
Hilimakora island	NS09-1.8A-2	N 1° 26′ 20.0″, E 97° 13′ 44.6″	+ 1.8	Fringing reef	16	66	δ^13^C shift
Hilimakora island	NS09-1.8A-5	N 1° 26′ 20.0″, E 97° 13′ 45.2″	+ 1.8	Fringing reef	18	77	δ^13^C shift
Hilimakora island	NS09-1.8A-6	N 1° 26′ 20.0″, E 97° 13′ 45.8″	+ 1.8	Fringing reef	18	74	δ^13^C shift
Sarangbaung island	NS09-M0.4A-3	N 1° 41′ 45.9″, E 97° 26′ 26.4″	− 0.4	Reef lagoon	17	59	Ba/Ca cyclicity

## Study site and coral drilling

Nias is located ~ 125 km offshore of the west Sumatran coast (1° N, 97.5° E) and experiences an equatorial climate with abundant rainfall year-round (~ 4000 mm annual average). Situated in the Indo-Australian monsoon region, Nias experiences associated shifts in surface winds, generally from the northwest in boreal winter and southeast in boreal summer. Aperiodic variations in the Indian Ocean Dipole (IOD) drive climate variability, with the positive mode marked by enhanced southeast winds, coastal upwelling and cooler SSTs, and reduced local rainfall^48^. The island’s terrain encompasses a mountainous inland area up to ~ 800 m in elevation surrounded by lowlands with dense vegetation. Rivers are perennially inundated leading to terrestrial discharge along coastal areas. A small concentration of rivers outflow along the northwest coast of Nias into the study area (Fig. 1). The dominant river is the Muzoi, with catchment headwaters extending ~ 40 km inland. Two smaller rivers, the Lafau and an unnamed river, have localized coastal plain catchments.

**Figure 2:**
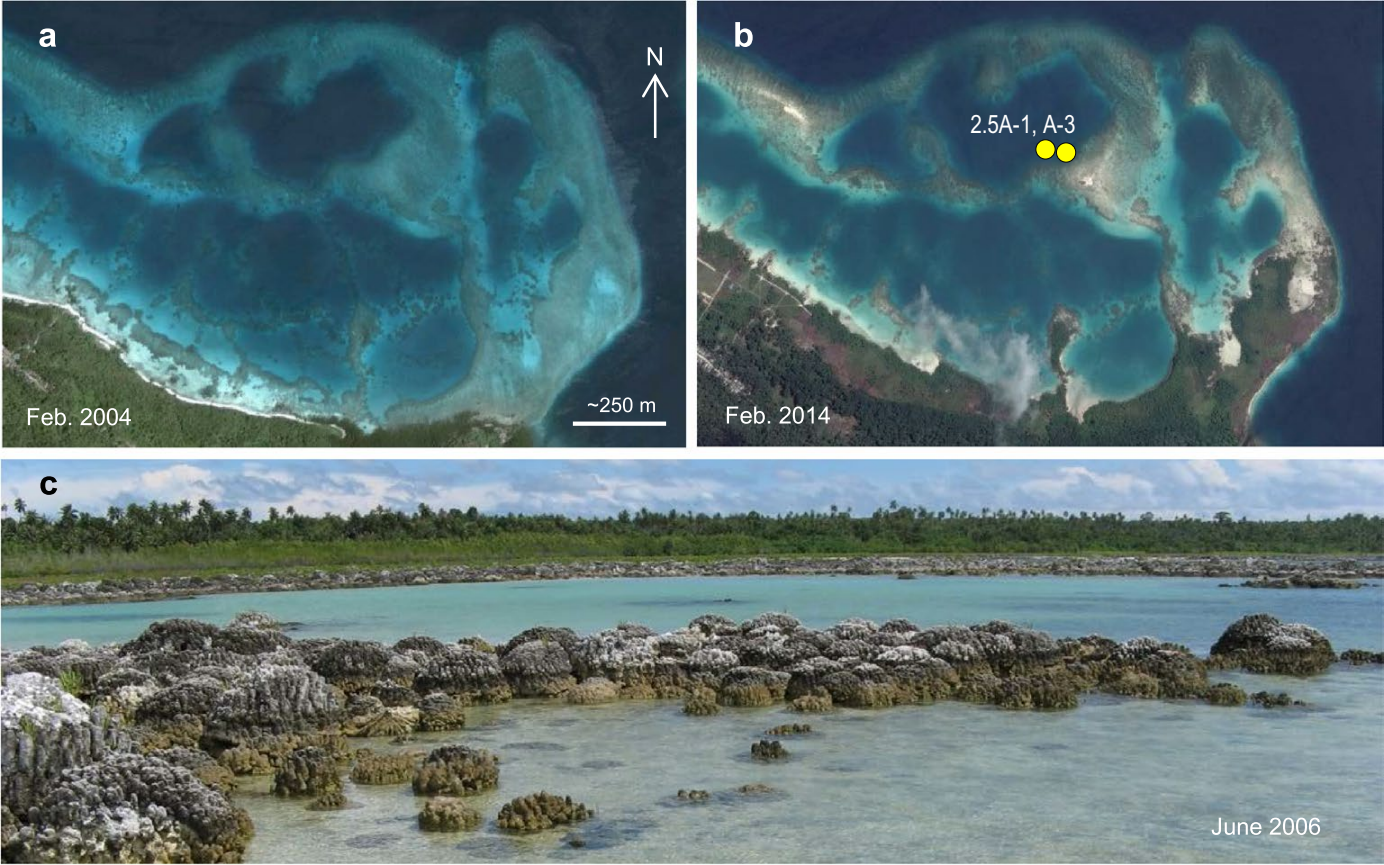
Images of coral drill-site 2.5A with the 2.5 m raised-reef lagoon. (**a**) Configuration of the deep-water lagoonal reef on 13 February 2004, about one year before the 28 March 2005 earthquake. (**b**) Raised reef on 17 February 2014 with exposure of new land and isolation of the shallowed lagoon due to uplift. Yellow circles show corals 2.5A-1 and 2.5A-3 drilled in May 2009. (**c**) 21 June 2006 image of raised *Porites* corals that colonized new seafloor created by subsidence prior to uplift. The *Porites-*dominated reef exposed along the coast (background) is adjacent to shallow, turbid lagoonal water. Map images are on Google Earth Pro 7.3.6.9345 (2022 version) at http://www.google.com/earth/index.html (accessed July 13, 2023).

Continuous GPS data and records of microatoll elevation were used to construct a contour map of coseismic uplift and subsidence along the Nias coastline produced by the 2005 earthquake^2^. Four years after the earthquake, we used the pattern of tectonic deformation to guide the drilling of submerged, dome-shaped *Porites* corals in May 23–29, 2009 at three sites^30^; two near the coast with strong uplift (+ 2.5 m, + 1.8 ± 0.18 m) and one offshore island control site with minor subsidence of − 0.42 ± 0.18 m (Fig. 1, Table 1). The + 2.5 m collection site (2.5A) is located within a broad fringing reef lagoon on the northwest corner of Nias, with large areas of the reef exposed post-earthquake (Fig. 2), proximal to the small river outlet. The + 1.8 m collection site (1.8A) is located 9 km to the east on the south side of Hilimakora Island, one of a series of small nearshore islands exposed to nearby runoff from the Muzoi and Lafau rivers. The control site, Sarangbaung Island (M0.4A), is located 20 km northeast of Nias, far from the influence of coastal rivers. The semi-diurnal tidal envelope along the coast of northwest Nias is microtidal with a maximum range of 1.2 m (Ref.^8^).

We analyzed cores from healthy coral colonies drilled vertically to a depth of 50–70 cm from the upper growth surface in close proximity to each other at the 2.5A (two corals) and 1.8A (three corals) uplift sites, and from one coral at the M0.4A control site offshore (Fig. 1, Supplementary Fig. 1). Previous work has shown that average annual coral extension rates, based on annual cycles of δ^13^C in coral skeleton that grew after the earthquake, and density band widths in x-radiographs, range from 16 to 23 mm/year at the 1.8A and M0.4A sites^30^, which is typical for massive *Porites* colonies growing in warm tropical settings^49^. In this study, we use annual cycles of Ba/Ca in coral skeleton that grew after the earthquake to estimate similar annual extension rates of 14–17 mm/year at the 2.5A and M0.4A sites (see “Methods”, Table 1, Supplementary Figs. 2–5).

## Results and discussion

### Behaviour of coral Ba, Mn and Y in the aftermath of the 2005 earthquake

Here, we explore the nature of post-earthquake changes in the new coral skeletal Mn/Ca, Y/Ca and Ba/Ca records and their links to altered environmental drivers, including terrestrial sediment delivery and reef-specific hydrographic factors (lagoonal reef versus fringing reef). Due to the pervasive year-round rainfall and lack of strong climatic events in northwest Nias, we attribute sustained shifts (> 1 year) in coral geochemistry across the time window of the coral cores (2000–2009.5) to earthquake-related environmental changes (see “Methods”). However, further validation of the coral geochemical response to earthquakes requires in situ records of the effect of river discharge on the water quality of the northwest Nias reefs, which are currently not available.

#### Post-earthquake enhancement of sediment delivery

**Figure 3:**
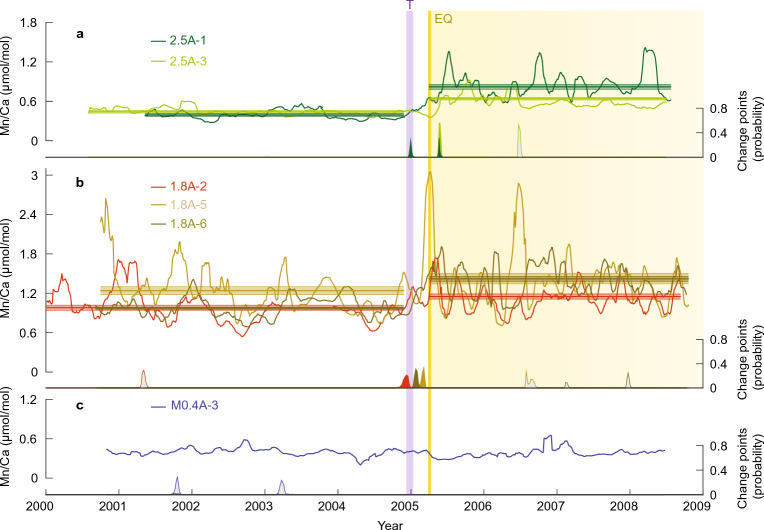
Coral Mn/Ca records before and after the March 2005 earthquake. Results are shown as 10-pt running means for the 2.5A and 1.8A uplift sites (**a**, **b**) and the M0.4A subsidence site (**c**) versus time. Yellow bar shows the earthquake; purple bar marks the December 2004 Indian Ocean tsunami. Profiles with significant pre- and post-earthquake differences in Mn/Ca are indicated by their non-overlapping means (horizontal lines) with shaded 95% confidence envelopes (1.96× standard error of the mean, see t-test results in Supplementary Table 1). Color-coded change points with posterior probability estimates^83^ confirm the reproducibility of a step-change earthquake signal at the 2.5A and 1.8A uplift sites. Change points occurring between 2004.5 and 2005.8 (inclusive of the tsunami and earthquake events) are filled, while those occurring outside of this range are grey. The reef-response interval following the earthquake at uplift sites is highlighted.

Average coral Mn/Ca values are highest at the 1.8A uplift site (1.2 μmol/mol) under the influence of runoff from the Muzoi and Lafau rivers (Fig. 3). By comparison, Mn/Ca values are much lower at the 2.5A uplift site (0.6 μmol/mol), proximal to the relatively small river, and lowest at the offshore subsidence site M0.4A (0.4 μmol/mol) with no riverine influence. Mn/Ca values for these corals fall within the range for *Porites* observed at other locations^45,50^. At the 2.5A uplift site, Mn/Ca shows sustained post-earthquake increases in both coral cores (2.5A-1, 2.5A-3) of 0.43 and 0.20 μmol/mol, respectively (Fig. 3, p < 0.0001 in Supplementary Table 1). Coral Mn/Ca at the 1.8A uplift site shows similar significant increases; 0.45 μmol/mol in coral 1.8A-6 and smaller increases of 0.17 and 0.18 μmol/mol in corals 1.8A-2 and 1.8A-5, respectively. Significant change points between 2004.9 and 2005.4 CE were identified across all five coral records for both uplift sites, in good alignment with the tsunami and earthquake (Fig. 3). In contrast, the M0.4A site shows negligible change in Mn/Ca (0.01 μmol/mol). Overall, these results indicate that coral Mn/Ca shows a significant step-change increase at the uplift sites ranging from ~ 0.17 to 0.45 μmol/mol, regardless of reef environment.

We propose that the increase in Mn/Ca is driven by a post-earthquake increase in sediment transport from land to sea. Earthquake and tsunami associated slope failure can remove vegetation and facilitate increased erosion, sediment transport, and discharge during high rainfall events. Previous work in wet sub-tropical Taiwan has documented increases in sediment delivery to coastal environments following large earthquakes^10,14^. The 2004 Boxing Day tsunami, which preceded the 2005 earthquake by three months, delivered waves up to 3.9 m in height at northwest Nias and left debris behind in trees^11^, and would have made the land surface susceptible to erosion. Indeed, the 2005 earthquake mobilized sediment from the mountainous regions to the coast, so much so that alterations in beach profile led to an increase in available nearshore sediment supply^3^. Furthermore, long-term shifts in coral Mn/Ca have been linked to mobilization of Mn associated with enhanced sediment delivery related to exposure and erosion of land surfaces associated with cyclone events and also catchment clearing^40,43^. Together, these findings indicate that earthquakes can abruptly alter nearshore reefs by enhancing sediment delivery to coastal regions, and coral Mn/Ca provides a useful proxy to monitor earthquake-induced changes in suspended sediment load.

**Figure 4:**
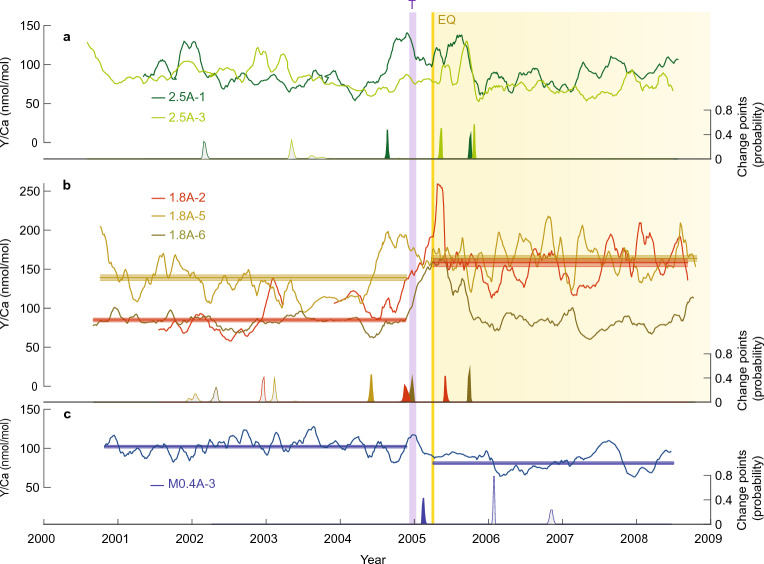
Coral Y/Ca records before and after the March 2005 earthquake. Results are shown as 10-pt running means for the 2.5A and 1.8A uplift sites (**a**, **b**) and the M0.4A subsidence site (**c**) versus time. Yellow bar shows the earthquake; purple bar marks the December 2004 Indian Ocean tsunami. Profiles with significant pre- and post-earthquake differences in Y/Ca are indicated by their non-overlapping means (horizontal lines) with shaded 95% confidence envelopes (1.96× standard error of the mean, see t-test results in Supplementary Table 1). Color-coded change points with posterior probability estimates^83^ confirm the presence of both transient and step-change earthquake signals at the 2.5A and 1.8A uplift sites. Paired change points for profiles 2.5A-1, 2.5A-3, 1.8A-2, and 1.8A-6 mark significant transient increases in Y/Ca. A change point also corresponds with the decrease in Y/Ca at subsidence site M0.4A-3. Change points occurring between 2004.5 and 2005.8 (inclusive of the tsunami and earthquake events) are filled, while those occurring outside of this range are grey. The reef-response interval following the earthquake at uplift sites is highlighted.

In addition to the changes in coral Mn/Ca, we document variations in coral Y/Ca associated with increased sediment delivery (Fig. 4). Corals A-2 and A-5 from the 1.8A uplift site, proximal to two rivers, have the highest mean Y/Ca values (136 nmol/mol), in contrast to M0.4A (91 nmol/mol) with no riverine influence (Supplementary Table 1), in line with other studies showing gradients in Y/Ca with increasing riverine influence^39,44^. The range of Y/Ca values at these sites is similar to that reported for *Porites* in the Great Barrier Reef^45^ (83 nmol/mol) and Puerto Rico^40^ (40 nmol/mol).

Y/Ca at the 1.8A uplift site shows a statistically significant step-change increase in corals A-2 (74 nmol/mol) and A-5 (25 nmol/mol) (Fig. 4, p < 0.0001 in Supplementary Table 1). Also, raised corals 1.8A-6, 2.5A-1 and 2.5A-3 show a prominent transient increase in Y/Ca around the time of the earthquake (Fig. 4). Furthermore, change point analysis reveals a transient increase in coral 1.8A-2 superimposed on the step-change increase in mean Y/Ca, with an initial rise in Y/Ca at 2004.9 CE and return to the post-earthquake mean at 2005.4 CE. Shared change points identified in corals 1.8A-6 and 1.8A-2 indicate that the transient increase in Y/Ca for two of the three cores at this site occurred between 2004.9 CE and 2005.7 CE. The subsidence site coral M0.4A-3 shows no transient peak associated with the earthquake, but rather a decrease in Y/Ca (26 nmol/mol) marked by a change point and step-change in post-earthquake mean.

Taken together, the results for the two uplift sites show either a sustained post-earthquake step-change increase in Y/Ca, or a transient increase in Y/Ca around the time of the earthquake. We suggest that these signals are linked to increased sediment availability, as Y/Ca has been shown to record changes in suspended sediment on annual to longer timescales related to large erosion events^39,45,47^. Indeed, during our initial visit to northwest Nias in June 2006, about 14 months after the earthquake, turbidity was particularly high in the + 2.5 m raised-reef lagoon offshore of Lahewa. Therefore, the tendency for transient increases in Y/Ca at the 2.5A lagoonal reef site could be due to tsunami, initially, followed by transient post-earthquake activation of the localised river sediment supply and wind-driven sediment resuspension in the shallow lagoon. Also, in June 2006 the *Porites* corals in the high uplift area were coated with green–brown algae. Post-earthquake decomposition of algal mats and development of reducing conditions could contribute to the pulse-like Y/Ca signature in coral cores at the 1.8 m and 2.5 m uplift sites, similar to changes observed in western Sumatra during a massive red tide^51^. The different nature of responses in coral Y/Ca, either transient or long term, suggests that within-reef differences in sediment availability and/or coral-colony specific factors could play a role in coral Y/Ca variability. The absence of an increase in Y/Ca at the offshore subsidence site supports the idea of increased sediment activation specific to raised-reef sites. In sum, the Mn and Y records both suggest that nearshore suspended sediment availability expands in the aftermath of coseismic uplift and alters the geochemical inventory of raised-reef environments in proximity to land*.*

#### Reef-specific earthquake-induced environmental changes

**Figure 5:**
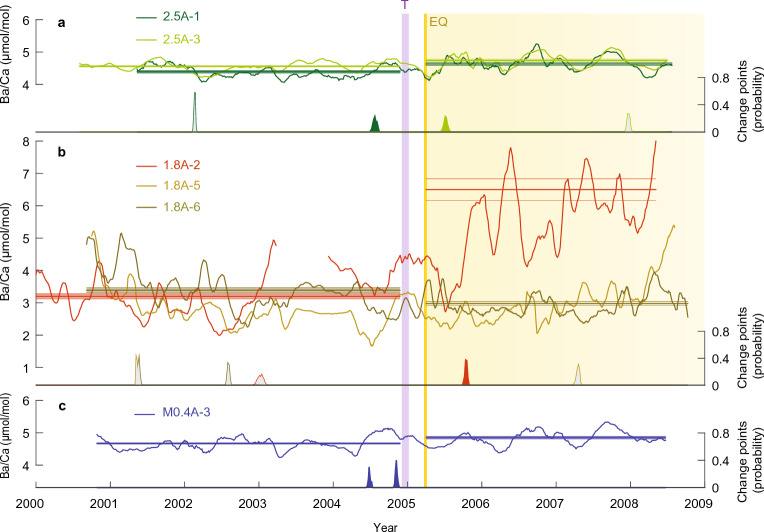
Coral Ba/Ca records before and after the March 2005 earthquake. Results are shown as 10-pt running means for the 2.5A and 1.8A uplift sites (**a**, **b**) and the M0.4A subsidence site (**c**) versus time. Yellow bar shows the earthquake; purple bar marks the December 2004 Indian Ocean tsunami. Profiles with significant pre- and post-earthquake differences in Ba/Ca are indicated by their non-overlapping means (horizontal lines) with shaded 95% confidence envelopes (1.96 × standard error of the mean, see t-test results in Supplementary Table 1). Color-coded change points with posterior probability estimates^83^ occurring between 2004.5 and 2005.8 (inclusive of the tsunami and earthquake events) are filled, while those occurring outside of this range are grey. The reef-response interval following the earthquake at uplift sites is highlighted.

In addition to changes in sediment delivery associated with uplift, we propose that different types of reefs (fringing versus lagoonal) experience divergent patterns of post-earthquake environmental change, as evident from the Nias coral Mn/Ca and Ba/Ca records (Fig. 5). Skeletal Ba/Ca values for lagoonal reef sites (2.5A, M0.4A) show a seasonal cyclicity with average values (4.5–4.8 umol/mol) similar to those in *Porites* exposed to upwelling of Ba-enriched waters^32^. In contrast, the coral Ba/Ca values for the 1.8A fringing reef site are lower on average (2.8–4.2 umol/mol), with annual peaks of varying intensity, similar to Ba/Ca records for sites proximal to rivers in Singapore with high suspended sediment loads^52^ (2–14 umol/mol). At the lagoonal sites, with uplift (2.5A) and subsidence (M0.4A), post-earthquake Ba/Ca increased by only 0.18–0.24 umol/mol (Supplementary Table 1). On the other hand, coral Ba/Ca at the fringing reef site (1.8A) shows a wide range of responses, including a particularly large increase in coral 1.8A-2 and a significant post-earthquake decrease in coral A-6. While the change point analysis highlights Ba/Ca responses occurring within the earthquake interval in individual records, they are not replicated by other records at the same site, or across sites, indicating a variable response of Ba/Ca to the 2005 earthquake.

These observations suggest that coral Ba/Ca has the potential to provide additional information about reef-specific impacts caused by earthquakes. In the wake of the 2004 tsunami, short-term increases in primary productivity^53,54^ could lead to pulse-like increases in Ba/Ca prior to the 2005 earthquake, followed by long-term increases in sediment resuspension and nutrients. Additionally, the 2.5A lagoonal reef site is proximal to a small river outlet, thus the long-term increase in coral Ba/Ca at this locality could be primarily driven by a mechanism specific to that reef. Distinct green–brown banding is present in the post-earthquake section of the site 2.5A drill-cores providing a visual indicator of earthquake-induced environmental stress and changes in primary productivity (Supplementary Figs. 3 and 4). Extensive shallowing of the raised-reef lagoon made the lagoon floor more susceptible to wind-induced sediment resuspension, and previous research has shown that accompanying release of sediment porewater nutrients drives algal blooms^55–57^. This increase in primary production would alter the Ba^2+^ inventory in the reef waters by barite formation, and resuspension of Ba-rich particles, as shown in previous studies^37,38^. Therefore, we propose that the post-earthquake increase in coral Ba/Ca at the + 2.5 m uplift site is related to increased lagoonal Ba^2+^ via wind-driven resuspension of Ba-rich particles, and partially to an increase in sediment delivery.

The sediment resuspension mechanism also could partly explain the increase in coral Mn/Ca at the 2.5A lagoonal site given that decaying organic matter produces reducing conditions leading to increases in porewater Mn^2+^ .^39,42^. Thus the post-earthquake increase in Mn/Ca might be related to northwest wind-driven remobilization of sediment porewater Mn^2+^, leading to seasonal increases in Mn^2+^ ^50,58^. Along the same lines, we attribute the post-earthquake increase in coral Ba/Ca at the offshore subsidence site (M0.4A) to increased wave-penetration across the lowered reef-crest and sediment resuspension in the deepened lagoon. The variable response of Ba/Ca at the 1.8A fringing reef site also indicates that coral Ba/Ca is influenced by local variations (i.e., sediment delivery) and/or coral-colony specific factors. The results highlight the need for multiple coral Ba/Ca records for each reef location to provide composite records that resolve changes in sediment delivery^35^.

Overall, these findings suggest that coral Mn/Ca, Y/Ca and Ba/Ca records can provide information on sediment delivery and reef-specific environmental change associated with earthquake events. In the case of the 2005 earthquake in Nias, the sediment load of rivers in the coseismically uplifted area evidently increased, and lagoonal reef settings, both raised and submerged, were impacted by wind- and wave-driven sediment resuspension and phytoplankton blooms. The results for the 2005 earthquake event, together with current knowledge about the history of great-earthquakes along the Sunda megathrust, raise the possibility that recurring cycles of interseismic subsidence and coseismic uplift could set the pace of reef growth and retreat on the outer-arc islands west of Sumatra.

### Coral reef response to great-earthquake cycles

Detailed studies of the growth-response of abundant massive *Porites* spp*.* microatolls to vertical crustal displacement during great-earthquake cycles have illustrated the influence of neotectonics on the natural dynamics of the West Sumatran coral reefs^22–26,59–63^. The new Nias coral geochemical records demonstrate that distinct environmental changes occurred in raised coral reef environments in the aftermath of the 2005 Nias-Simeulue earthquake. Here, we explore the potential role of recurrent earthquake-induced environmental impacts in shaping the coral community structure and species composition of the outer-arc island fringing reefs. Notable in this regard, the diversity of scleractinian coral species along the west coast of Sumatra is the lowest in Indonesia^64^. Also, recent surveys show that the island fringing reefs offshore at Nias, Simeulue, and the Mentawai Islands are dominated by stress-tolerant coral species such as *Porites, Pavona* and *Psammocora* with massive and sub-massive growth forms^65^.

The available observational data on the pattern of coral damage in the aftermath of the 2005 earthquake at raised-reef sites along the northwest coast of Nias, including our study area, illustrate how earthquakes can alter the species composition of coral reefs. Post-earthquake surveys found a 50% decrease in live coral cover and a shift from branching to massive growth-forms as the dominant surviving coral group due to the vulnerability of the branching morphology to physical damage^66^. Our observations in May 2009 confirm that massive *Porites* colonies were the dominant survivors at our three study sites. Only partial recovery of coral cover had occurred on submerged sections of the Nias raised reefs by December 2015, ~ 10.5 years after the earthquake, in part because of further loss of live branching coral caused by seismic shaking during two massive strike-slip earthquakes (M_w_ 8.6 and 8.2) centred ~ 450 km west of Nias on 11 April 2012 ^67^. This post-seismic pattern of species-specific survival is analagous to well-documented reef damage associated with hurricanes, which destroy shallow-water branching corals and increase the relative abundance of encrusting and massive-shaped colonies^68,69^.

Coral reproduction and recruitment following abrupt disturbances is critical in setting the course of recovery for coral reef systems^69–71^. Availability of coral larvae and successful settlement and survival of recruits are critical factors governing this process^71^. The initial life stages of scleractinian corals can be negatively impacted by water quality; changes in sediment, salinity and dissolved inorganic nutrients can reduce gamete production, fertilisation, and embryo development^71,72^. Furthermore, the density of spawning adult coral colonies, specific to each species, is important for fertilisation success^71^. Low densities of reproductive branching corals post-earthquake can constrain fertilization success since the majority of viable larval recruitment occurs close to the reproductive source^71,73^. Additionally, any potential larval recruitment from undamaged reefs outside the earthquake rupture zone may be limited due to competiton for space by pioneering massive corals^70^. Consequently, the size of a surviving massive coral population may be enhanced by local larval input and preferential recruitment following an earthquake, thus increasing the likelihood of their presence in successive generations^74^.

**Figure 6:**
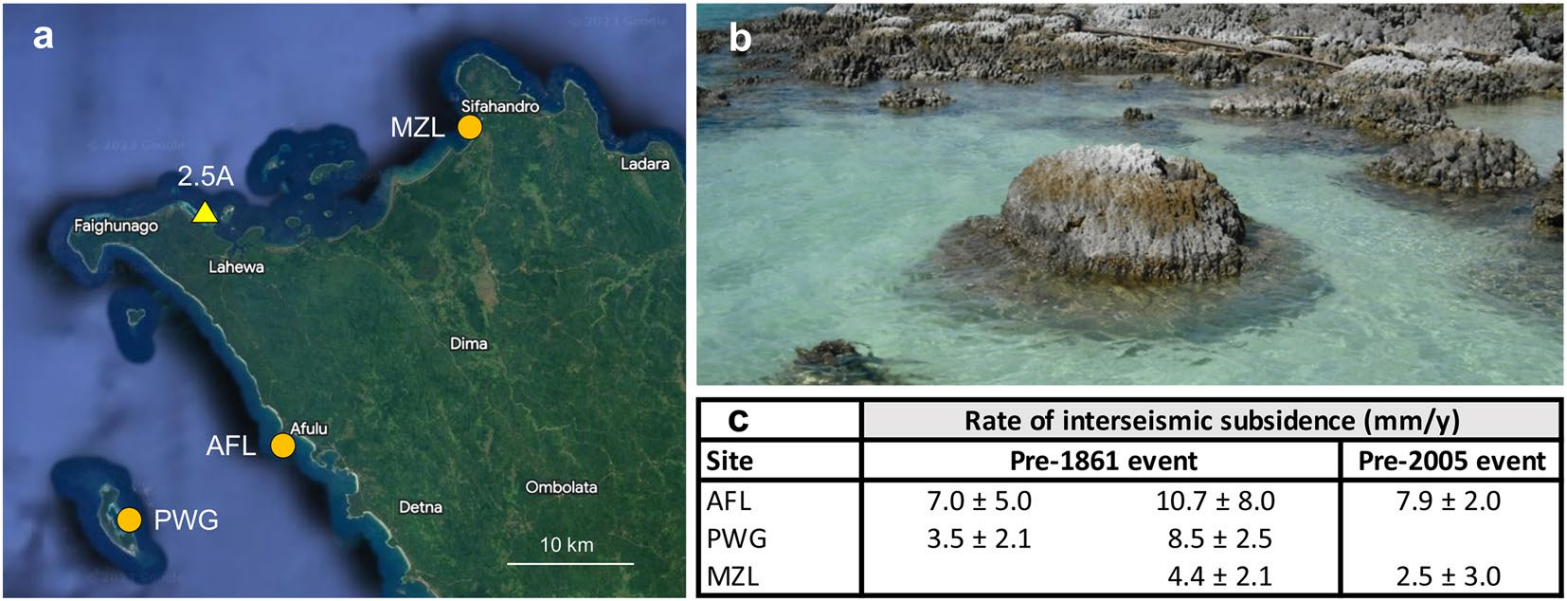
Coral reef submergence and expansion in northwest Nias prior to megathrust earthquakes. (**a**) Location of coral drill-site 2.5A (this study) and *Porites* microatoll records AFL, PWG and MZL (Ref.^25^) that document interseismic subsidence prior to the 1861 and 2005 earthquakes. (**b**) 23 May 2009 image of *Porites* corals at the 2.5A uplift site that colonized new seafloor created by crustal subsidence before the 2005 earthquake. Foreground shows regrowth atop a dead *Porites* colony as it resubmerged during the decades leading-in to the 2005 earthquake. (**c**) Summary of subsidence rates recorded by microatolls AFL, PWG and MZL^25^. AFL and PWG recorded increased subsidence during the decades immediately before the 1861 earthquake, promoting reef expansion. Map image is for April 2023 on Google Earth Pro 7.3.9345 (2022 version) at http://www.google.com/earth/index.html (accessed June 15, 2023).

**Figure 7:**
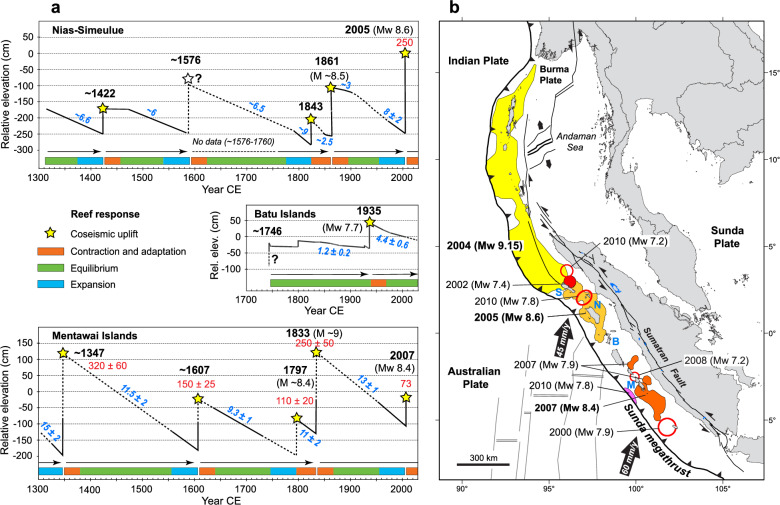
Temporal and spatial influence of great-earthquake cycles on coral reefs along the Sunda megathrust. (**a**) Schematic summary of earthquake cycles and coral reef response over the last seven centuries. Maximum coseismic uplift values are in red (± 2σ) and interseismic subsidence rates are in blue (in mm/y, ± 2σ); dotted lines are inferred. Color-coded bars show schematic three-phase reef response to culminating earthquakes and interseismic subsidence (orange bars indicate nominal 30 year windows for full post-earthquake reef recovery). The Nias-Simeulue example is a composite microatoll elevation record for Simeulue^24^ (Bunon village, 1311–1576 CE) and northern Nias^25^ (sites AFL, MZL, PWG; 1800–1861 CE, 1960–2000 CE). Batu Islands is a composite for south Tanabala island showing a rare locality with long-term tectonic stability^60,63^. Mentawai Islands shows strong earthquake cycles at Bulasat, South Pagai island^22^. (**b**) Spatial distribution of crustal ruptures for recent large earthquakes (> M_w_ 7) along the Sunda megathrust since 2000 CE. Red circles indicate estimated rupture areas. Blue letters show the approximate locations of the earthquake cycle time-series for Nias-Simeulue (N, S), Batu Islands (B) and the Mentawai Islands (M). Map is adapted from Natawidjaja et al.^23^ with rupture areas and magnitudes from Briggs et al.^2^, Chlieh et al.^85^, Konca et al.^86^, Meltzner et al.^62^, Hill et al.^87^, and references therein.

Based on these results, we suggest that the great-earthquake cycle in West Sumatra could serve to scale-up the localized environmental and ecological effects of neotectonics on coral reefs to the regional level (Figs. 6, 7). Studies of the growth-response of *Porites* microatolls to tectonic motion have revealed the repetition of great-earthquake cycles along the Sunda megathrust over the last ~ 700 years, with century-scale periods of interseismic subsidence terminated by coseismic uplift^22–26^. Our photographs of the raised reefs of northwest Nias following the 2005 earthquake show that tectonic subsidence, and reef expansion via coral colonization of new seafloor, was well underway during the decades prior to the 2005 earthquake (Figs. 2, 6). This observation is consistent with microatoll elevation data for northwest Nias showing interseismic subsidence leading-in to 2005, and also before the 1861 Nias earthquake^25^ (Fig. 6). These long intervals of interseismic quiescence provide stable environmental conditions for coral reef expansion before the system abruptly switches to post-seismic adaptation.

Figure 7 provides an overview of the spatial and temporal histories of interseismic subsidence and coseismic uplift along the Sunda megathrust. In general terms, we propose a three-phase coral reef response within each great-earthquake cycle: (1) post-earthquake reef contraction and adaptation; (2) slow century-scale subsidence and ecological equilibrium; and (3) reef expansion leading up to the final major earthquake. However, the spatial and temporal characteristics of each great-earthquake cycle are influenced by the tectonic status of different sectors along the Sunda megathrust. For instance, the Mentawai Islands and Nias-Simeulue sectors have broadly aligned and regular earthquake cycles lasting about 200 years^22–26^ (see Fig. 7). In contrast, the Batu Islands sector exhibits subdued tectonic activity compared to neighbouring sectors^60,63^. The records also indicate that the massive 2004 and 2005 earthquakes marked the culimination of the most recent earthquake cycle in the northernmost Sumatra–Andaman Island sector^24,25^, where the reef adaptation process is presumably well underway. However, the ensuing rupture sequence in the Mentawai Islands sector remains incomplete, increasing the likelihood of further coseismic uplift and reef adaptation^22^. Consequently, the current scenario is one of disparate tectonic and ecological backdrops, the net effect of which is to create a heterogeneous mosaic of reef responses across the region.

Among the implications, our findings highlight the need to consider the evolving tectonic, environmental and ecological status of the Sunda megathrust in assessments of anthropogenic degradation on the outer-arc island fringing reefs of West Sumatra. The integrated coral geochemical records and great-earthquake time-series indicate that most of the coral communities are currently at an unsual adaptational juncture. While the immediate loss of coral cover following coseismic upheaval is clear, the post-seismic environmental impacts could alter coral reef community structures for years. For instance, prolonged periods of elevated turbidity reduce light availability and suppress coral growth via reduced photosynthetic rates, with species-specific tolerance responses^75^. And shifts in primary productivity can lead to infestation of corals by endolithic algae, further affecting coral growth and survival^1,7,8^. Understanding the interplay between earthquakes and the variable baseline of coral reef health will be critical to quantify the broader impacts of human agency.

## Conclusions

New coral geochemical records for the 2005 Nias-Simeulue earthquake in West Sumatra provide valuable archives of earthquake-induced shifts in coral reef environmental conditions. Specifically, skeletal Mn/Ca reflects changes in sediment delivery at uplifted reef sites, regardless of reef type. The increase in sediment delivery associated with the earthquake is tied to changes in riverine geomorphology and reef-specific changes brought about by vertical displacement. Additionally, skeletal Y/Ca and Ba/Ca support the idea of changes in sediment delivery to reef environments in the aftermath of earthquakes. Raised-reef lagoonal Mn/Ca and Ba/Ca records highlight the role of shoaling and changes in local hydrography on resuspension of sediments and primary productivity. Our analysis of the 2005 event indicates that abrupt shifts in coral reef environments can alter the community structure of coral reefs, and raises the possibility that recurrent great-earthquake cycles along the Sunda megathrust have played a role in shaping the low species diversity of corals on the West Sumatran reefs. While our focus here is on the outer-arc island fringing reefs along the Sunda megathrust, the earthquake cycle reef-response concept could be applied to improve our understanding of the community status of coral reefs along subduction zone margins elsewhere in the tropics.

## Methods

### Coral sample preparation and trace element analysis

Coral cores were longitudinally slabbed into 7-mm thick slices and X-rayed in order to identify a maximum growth axis and develop a basic chronology for each coral record (Supplementary Figs. 2–5). The slabs were cleaned using a hand-held ultrasonicator probe in Milli-Q water and dried overnight in an oven at 40 °C. After removal of material for stable isotope analysis^30^, a 25 × 95 mm (maximum) section was cut from the coral slice to fit within the sampling cell for analysis by laser ablation inductively coupled plasma mass spectrometry (LA-ICP-MS) at the Research School of Earth Sciences, the Australian National University. Each section was vigorously cleaned using a hand-held ultrasonicator in Milli-Q water and placed in an oven overnight to dry at 40 °C. Laser scan-paths were selected to parallel the major growth axis of the coral and the stable isotope sampling path across at least nine years of coral growth to capture pre- and post-earthquake elemental variability.

The LA-ICP-MS laboratory procedures for analysis of trace elements in corals have been described in detail^42,76,77^. Briefly, the LA-ICP-MS system uses a 193 nm ArF excimer laser coupled to a Varian 820 ICP-MS to quantify trace element intensities. Ablated material was delivered to the ICP-MS via a mixed stream of Ar and He (Ref.^78^). Prior to analysis, laser tracks were pre-ablated to remove surface contaminants using a large 100 um × 700 um rectangular spot, with the laser set at 20 Hz and 100 mJ. For the analytical work, ablation analysis was conducted in two consecutive traverses of the sample: major trace element analysis (Ba, Mn) using a 50 × 500 um spot size followed by trace element analysis (Y) using a 100 × 500 um spot size, as outlined in Ref.^42^. The ablation analyses were carried out at a 40 um/s scan speed, 5 Hz repetition rate, 50 mJ energy, and using a 50% partially reflecting mirror. Measurements on standards were made at the beginning and end of each analysis section; the NIST 614 silicate glass certified reference material and an in-house pressed coral powder standard were used to normalize Y and Mn, and Ba, respectively^42,79^. The normalized data were smoothed with a 10-point averaging box filter to remove high frequency variation due to instrumental instability and counting statistics (Supplementary Figs. 6–8). All reported Y/Ca values are at least one order of magnitude greater than the instrument detection limit^42^.

We note that in some studies a stepwise pretreatment with intensive cleaning procedures (i.e., hydrogen peroxide rinses) has been employed for coral trace element analyses to ensure that the measured Mn and Y has substituted for Ca in the aragonite lattice, rather than reflecting organic phases or Mn oxide coatings^41,43,80^. We follow the method of Ref.^42^, which incorporates several pre-ablation steps to ensure minimal surface contamination. To assess the Mn signal we acquired Mn data from both analyses (major and trace element) along the same track in coral core 2.5A-1. We see similar post-earthquake increases in Mn in both analyses, which suggests the Mn signals are lattice bound (Supplementary Fig. 6). Also, there is a steep rise in Mn/Ca at the end of both records that is mostly restricted to skeletal elements that were surrounded with live coral tissue at the time of collection.

### Coral core chronologies

We use the approach of Gagan et al.^30^ to generate age models for the elemental records for three previously published coral cores at the 1.8A uplift site. In this earlier study, the position of the step-change in skeletal δ^13^C due to uplift marks the position (and date) of the earthquake within each coral core at the 1.8A site. This chronological ‘anchor point’ was then used to estimate average annual coral extension rates. In most cases, the position of the earthquake signal within the coral cores is also marked by a slight change in skeletal density evident in X-radiographs. The methodology indicates reasonable growth rates of 16–18 mm/year for the three 1.8A corals anaysed for the present study (Table 1).

While this approach is valid for the 1.8A site, there is no clear signal in coral δ^13^C at the 2.5A uplift site, presumably due to higher water column turbidity post-earthquake, which masked the effect of increased light intensity due to uplift^30^. Additionally, the juvenile coral M0.4A-3 does not show a distinct change (decrease) in δ^13^C associated with subsidence and a decrease in light intensity, possibly due to ontogenetic effects^81^. In these cases, we use coral Ba/Ca cyclicity alongside skeletal density patterns in x-radiographs and coral core features to constrain the earthquake window. At the 2.5A and M0.4 sites, coral Ba/Ca shows a distinct seasonal cyclicity, linked in other regions to seasonal upwelling^31,32^ (Supplementary Figs. 3–5). We align high Ba/Ca peaks in the records to mid-October, around the end of the southeast monsoon when wind-driven upwelling and low sea surface temperatures reflect maximum shoaling of the thermocline^82^, and low Ba/Ca peaks to mid-April, when upwelling is weak near the end of the northwest monsoon season.

Using this approach, the March 2005 earthquake event is at 59–63 mm depth within the 2.5A and M0.4A coral cores (Table 1), which equates to reasonable growth rates of 14–17 mm/year over the post-earthquake interval (with the core-top set to May 2009). At the 2.5A site, the depth of the Ba/Ca-assigned earthquake event within the coral cores aligns with the base of a distinctive green–brown discoloration, distinctive high-density bands, and a shift to higher mean Ba/Ca (Supplementary Figs. 3, 4). We note that the juvenile coral M0.4A-3 grew more slowly after the earthquake (59 mm) compared to the other three M0.4A corals analyzed by Gagan et al.^30^, where a step-change decrease in skeletal δ^13^C due to coseismic subsidence occurs at 82–86 mm depth within the cores.

The primary goal of our study is to examine broad geochemical changes before and after the 2005 earthquake, thus basic age models are suitable for application to the ~ 9-year coral records. To quantify the post-earthquake geochemical response, averages were calculated for the coral growth intervals before (2000–2004.9) and after the earthquake (2005.25–2009) at each site. For site 1.8A, we show the extended dataset for coral core 1.8A-2 (Supplementary Fig. 7; 150–180 mm) to demonstrate the longer-term stability of the geochemical baseline prior to the earthquake, but only present the 0–150 mm interval in Fig. 3 to be consistent with other cores from this site. We performed t-tests to determine the significance of any post-earthquake geochemical shift using pre- and post-earthquake averages based on 4–5 years of coral growth (Supplementary Table 1). The t-test calculations exclude elevated Ba/Ca and Mn/Ca values that occur within (or immediately below) skeleton surrounded by live coral tissue at the time of collection. Specifically, at site 1.8A we see significantly elevated Ba/Ca values proximal to the tissue layer (Supplementary Fig. 7), which is consistent with previous studies of coral Ba/Ca (tenfold higher below tissue layer^45^. The t-test analysis examines long-term shifts in geochemical records and thus does not include the Y/Ca records for corals 2.5A-1, 2.5A-3 and 1.8A-6 as they show transient increases with the earthquake.

In addition to the t-tests, a Bayesian change point algorithm^83^ was applied to the coral datasets to statistically test for the occurrence of change points associated with the December 2004 tsunami and 2005 earthquake. The algorithm is applied to normalized Mn/Ca, Y/Ca, and Ba/Ca records for each coral across all three sites. Analyses exclude data from within the tissue layer (upper 10 mm) of each coral. A piecewise constant regression model is inferred by the algorithm to determine the posterior probability of a change point. Input parameters for the change point analysis include a minimum of 35 data points between change points (d_min_), as well as a maximum number of change points permitted per solution (k_max_), where 1000 independently sampled soutions are performed on each record. To define step-change earthquake signals, k_max_ was set to two for the Mn/Ca and Ba/Ca records. To allow for identification of the onset and termination of transient earthquake signals in the Y/Ca records, k_max_ was set to three. The change point probability density curves represent the probability of a change point occurring at each point in time—a result of the 1000 Bayesian solutions for each record. The presence of an earthquake signal is defined by change points that occur within the age-range of 2004.5–2005.8 CE. This 15-month window, including both the December 2004 tsunami and the March 2005 earthquake, allows for an estimated uncertainty of ± 0.5 years in the coral core chronologies, and the observation that coseismic shifts in the geochemical records are not ‘knife sharp’ when plotted as a function of time. The smoothing effect may reflect the duration of environmental change and the attenuation of geochemical signals in corals by skeletogenesis throughout the depth of the living coral tissue layer^84^.

### Supplementary Information

Supplementary Information.

## Data Availability

The coral geochemical data presented in this study are archived at the NOAA-NCEI World Data Service for Paleoclimatology.
