# Supplementary material for: Coral geochemical response to uplift in the aftermath of the 2005 Nias–Simeulue earthquake
Source: Sci Rep. 2024 Apr 15;14:8686. doi: 10.1038/s41598-024-57833-1 (PMC11018842; doi:10.1038/s41598-024-57833-1)
Supplement: Supplementary file 1 — Supplementary Information. [file 41598_2024_57833_MOESM1_ESM.pdf]

Supplementary Information

Coral geochemical response to uplift in the aftermath of the 2005 Nias-Simeulue earthquake

Sindia M. Sosdian[1,2,*], Michael K. Gagan[2,3,4], Danny H. Natawidjaja[5], Alena K. Kimbrough[2,3], Bambang W. Suwargadi[6], Hamdi Rifai[7], Heather Scott-Gagan[2], Dudi Prayudi[6], Imam Suprihanto[6], Wahyoe S. Hantoro[3,4,6†]

[1]School of Earth and Ocean Sciences, Cardiff University, Cardiff, CF10 3AT, United Kingdom
[2]Research School of Earth Sciences, The Australian National University, Canberra, ACT 2601, Australia
[3]School of Earth, Atmospheric and Life Sciences, University of Wollongong, Wollongong, NSW 2522, Australia
[4]School of Earth and Environmental Sciences, The University of Queensland, St Lucia, QLD 4072, Australia
[5]Research Center for Geological Disaster, National Research and Innovation Agency (BRIN), Bandung 40135, Indonesia
[6]Research Center for Geotechnology, Indonesian Institute of Sciences (LIPI), Bandung 40135, Indonesia
[7]Department of Physics, Universitas Negeri Padang, Padang 25131, Indonesia
[†]Deceased

*Corresponding author: sosdians@cardiff.ac.uk

Supplementary Figures

Figure 1: Underwater photos of *Porites* spp. corals drilled at sites NS09-2.5A, NS09-1.8A and NS09-M0.4A.

Figure 2: X-ray positive images for coral drill-cores NS09-1.8A-2, NS09-1.8A-5 and NS09-1.8A-6.

Figure 3: Plain light photo, X-ray positive image and Ba/Ca age model for coral drill-core NS09-2.5A-1.

Figure 4: Plain light photo, X-ray positive image and Ba/Ca age model for coral drill-core NS09-2.5A-3.

Figure 5: X-ray positive image and Ba/Ca age model for coral drill-core NS09-M0.4A-3.

Figure 6: LA-ICP-MS Ba/Ca, Mn/Ca and Y/Ca data profiles for drill-cores NS09-2.5A-1 and NS09-2.5A-3.

Figure 7: LA-ICP-MS Ba/Ca, Mn/Ca and Y/Ca data profiles for drill-cores NS09-1.8A-2, NS09-1.8A-5 and NS09-1.8A-6.

Figure 8: LA-ICP-MS Ba/Ca, Mn/Ca and Y/Ca data profiles for drill-cores NS09-M0.4A-3.

Supplementary Data

Table 1: T-test results for pre- and post-earthquake elemental ratios in the Nias corals.

Supplementary References

Supplementary Figures

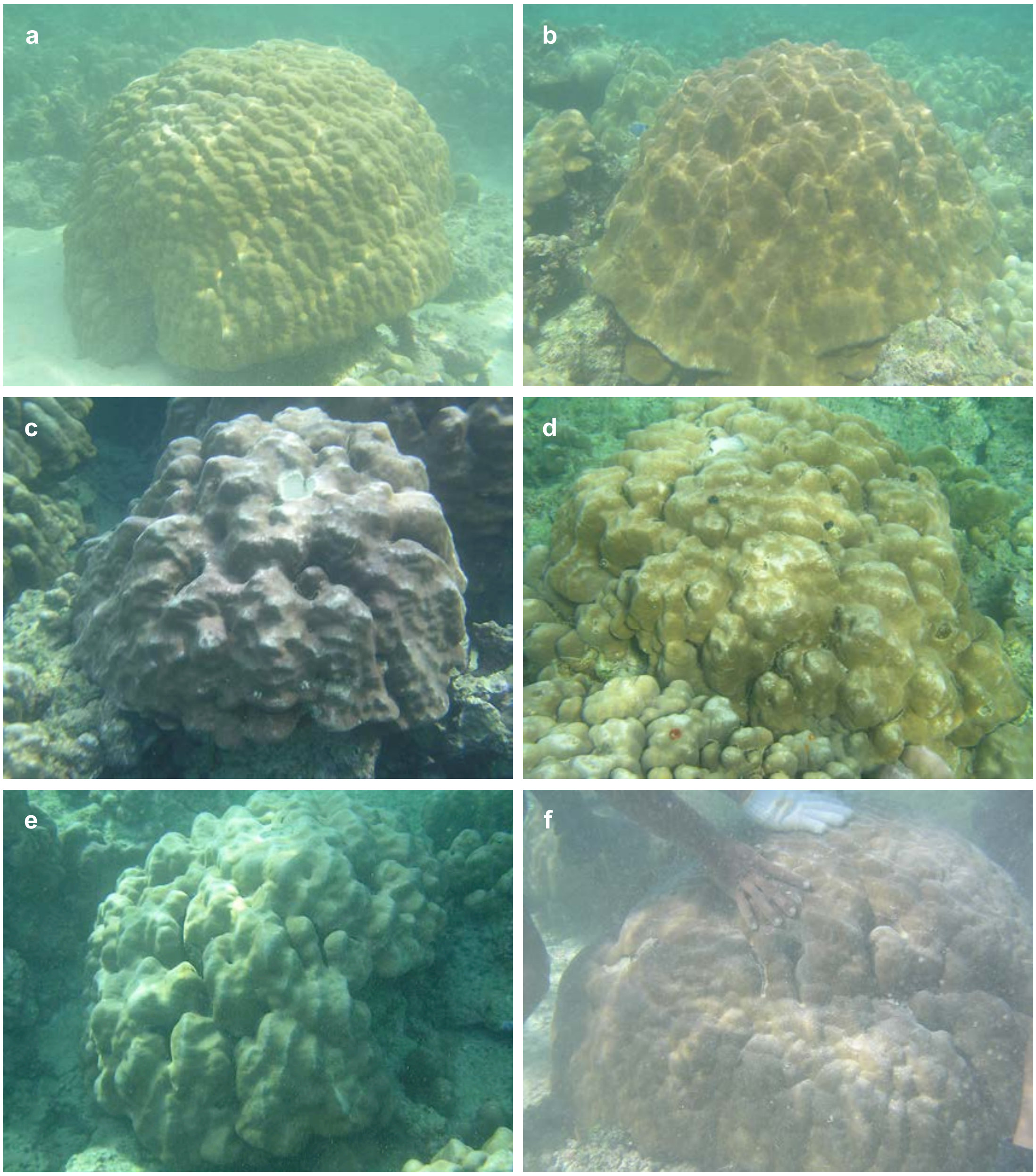

Figure 1 | Underwater photos of *Porites* spp. corals drilled at sites NS09-2.5A, NS09-1.8A and NS09-M0.4A. Corals 2.5A-1 (**a**) and 2.5A-3 (**b**) drilled on 20 May 2009 in turbid water in the shallow platform reef lagoon created by 2.5 m coseismic uplift west of Lahewa Harbour. Corals 1.8A-2 (**c**), 1.8A-5 (**d**) and 1.8A-6 (**e**) drilled on 24-25 May 2009 along the fringing reef at Hilimakora Island with 1.8 m coseismic uplift. Coral M0.4A-3 (**f**) drilled on 27 May 2009 on the offshore fringing reef lagoon at Sarangbaung Island with 0.4 m coseismic subsidence.

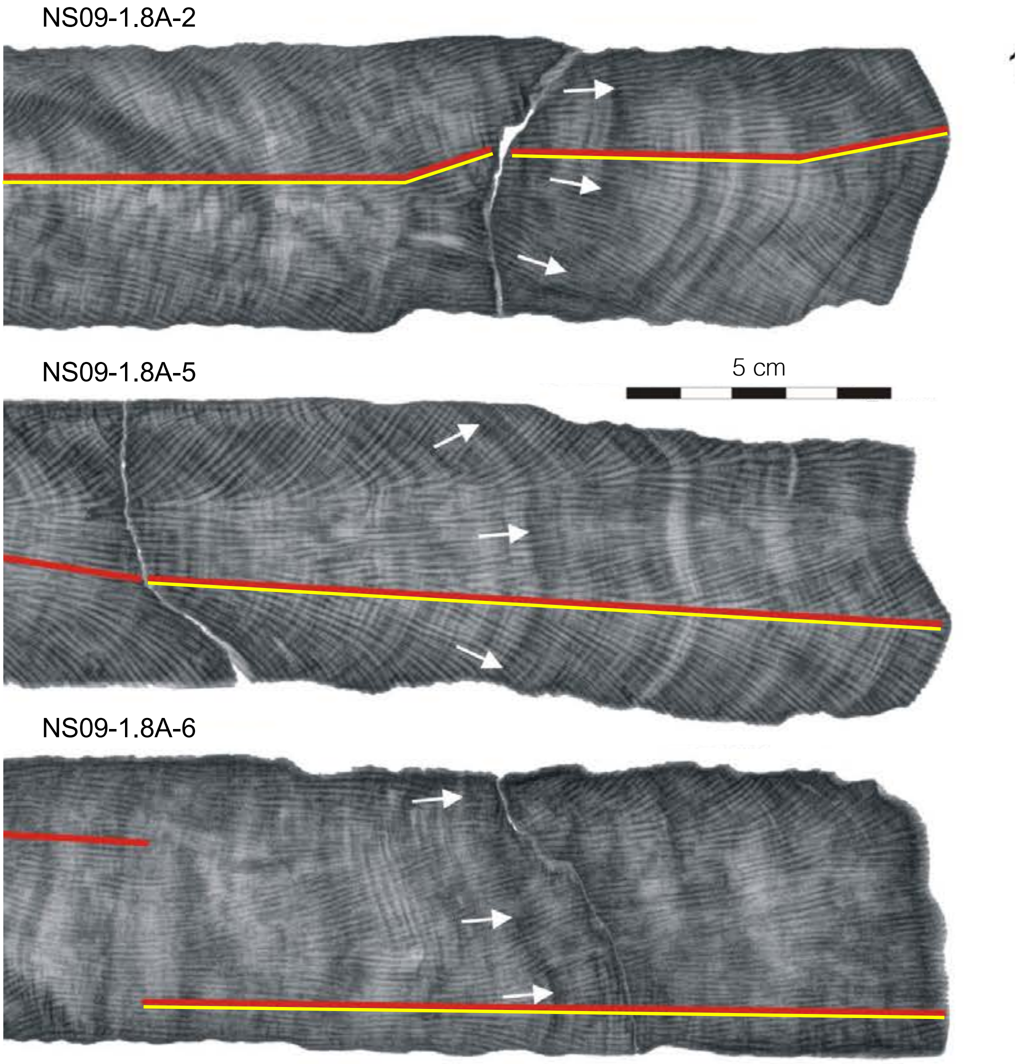

Figure 2 | X-ray positive images (darker = higher density) for coral drill-cores NS09-1.8A-2, NS09-1.8A-5 and NS09-1.8A-6 (adapted from Gagan et al. 2015). The corals were drilled underwater on the fringing reef at Hilimakora Island that was raised by 1.8 m during the 28 March 2005 earthquake (Briggs et al. 2006). Arrows in each core mark the depth of a slight increase in skeletal density following the earthquake. Red lines show sampling transects used for stable isotope analysis (Gagan et al. 2015). Yellow lines show the ~150-mm lengths of the LA-ICP-MS sampling tracks positioned alongside the stable isotope transects.

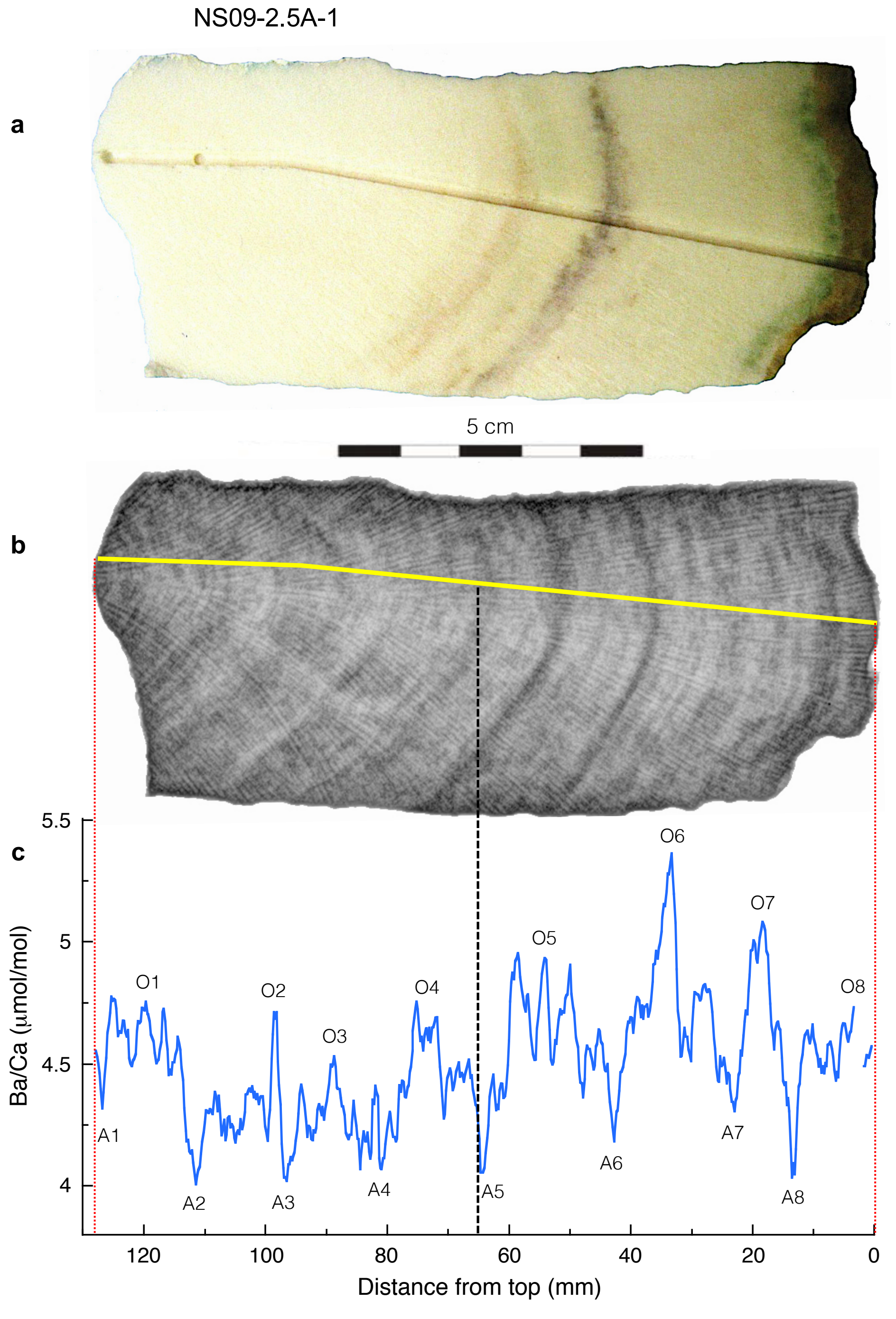

Figure 3 | Plain light photo, X-ray positive image and Ba/Ca age model for coral drill-core NS09-2.5A-1. (**a**) Photo of the coral slab for *Porites* sp. coral 2.5A-1 drilled underwater in the shallow lagoon of the raised reef west of Lahewa Harbour. The coral was raised by 2.5 m during the 28 March 2005 earthquake (Briggs et al. 2006) and shows post-earthquake skeletal discolouration and distinctive green-brown banding. (**b**) X-ray positive image (darker = higher density) of the coral slab. Yellow line shows the 128-mm length of the LA-ICP-MS sampling track. (**c**) Seasonal cycles of skeletal Ba/Ca (5-pt running means) used to develop the age model for coral 2.5A-1. The chronology is based on linear interpolation between tie-points assigned to mid-April (low Ba/Ca) and mid-October (high Ba/Ca) (see Methods). Black dashed line marks approximate depth to the March 2005 earthquake.

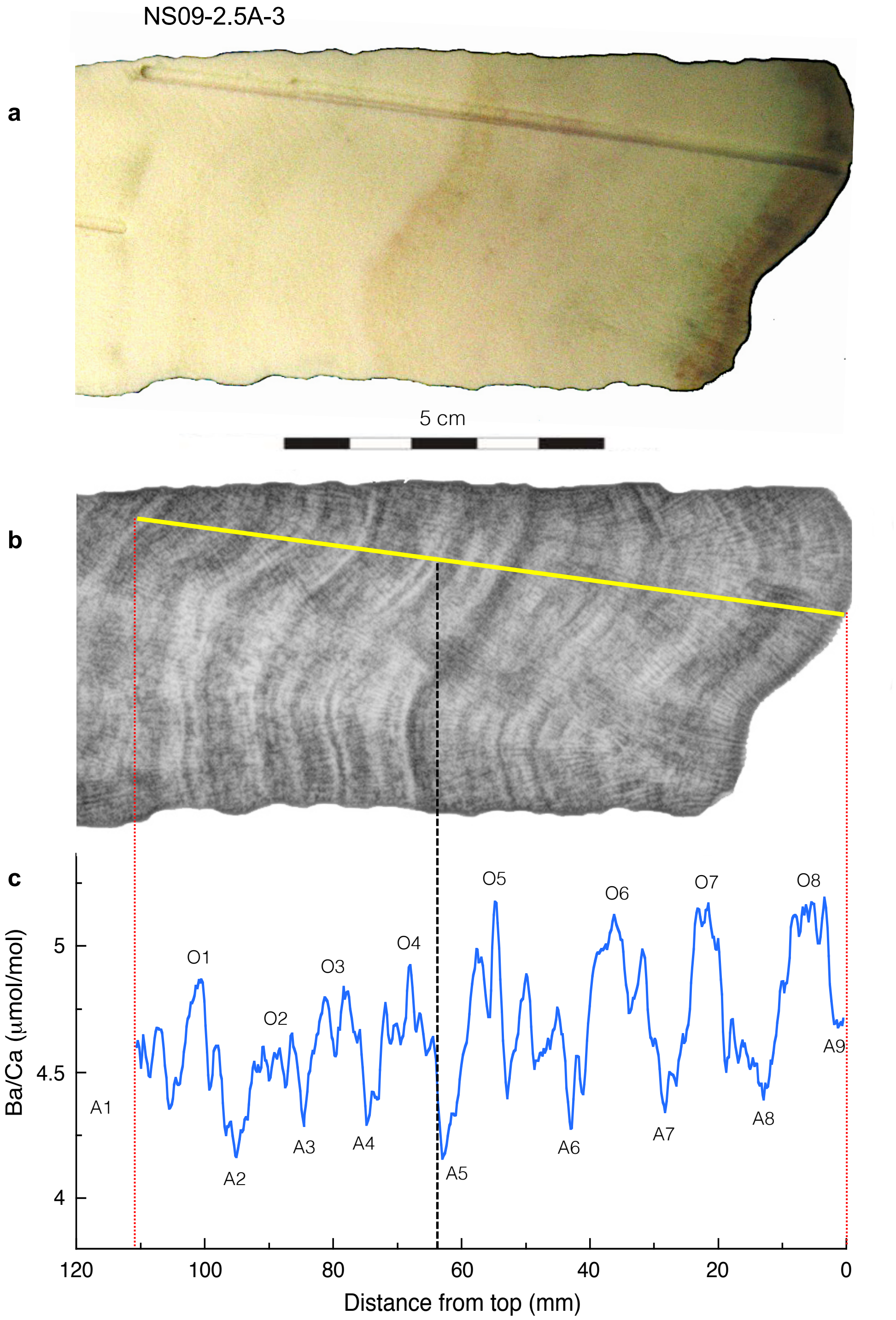

Figure 4 | Plain light photo, X-ray positive image and Ba/Ca age model for coral drill-core NS09-2.5A-3. (**a**) Photo of the coral slab for *Porites* sp. coral 2.5A-3 drilled underwater in the shallow lagoon of the raised reef west of Lahewa Harbour. The coral was raised by 2.5 m during the 28 March 2005 earthquake (Briggs et al. 2006) and shows post-earthquake skeletal discolouration. (**b**) X-ray positive image (darker = higher density) of the coral slab. Yellow line shows the 111-mm length of the LA-ICP-MS sampling track. (**c**) Seasonal cycles of skeletal Ba/Ca (5-pt running means) used to develop the age model for coral 2.5A-3. The chronology is based on linear interpolation between tie-points assigned to mid-April (low Ba/Ca) and mid-October (high Ba/Ca) (see Methods). Black dashed line marks approximate depth to the March 2005 earthquake.

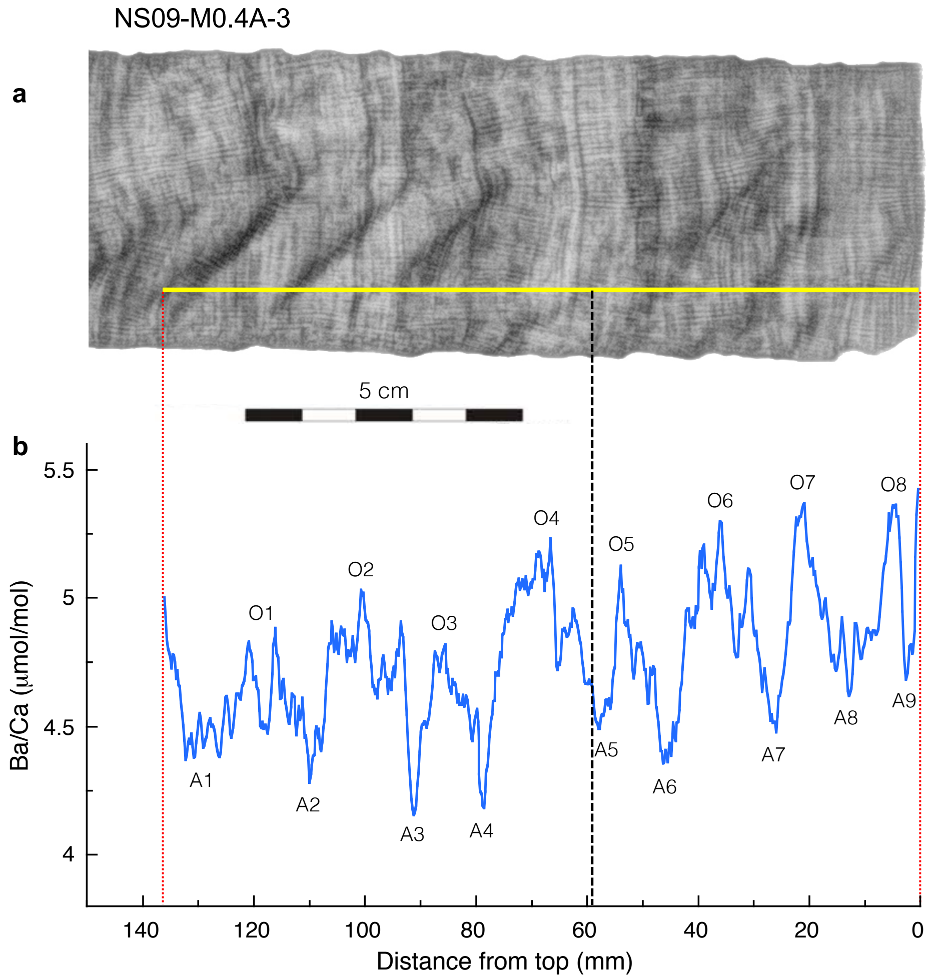

Figure 5 | X-ray positive image and Ba/Ca age model for coral drill-core NS09-M0.4A-3. (**a**) X-ray positive image (darker = higher density) of *Porites* sp. coral M0.4A-3 drilled underwater in the lagoon of the submerged reef at Sarangbaung Island. The coral subsided by 0.4 m during the 28 March 2005 earthquake (Briggs et al. 2006). Yellow line shows the 137-mm length of the LA-ICP-MS sampling track. (**b**) Seasonal cycles of skeletal Ba/Ca (5-pt running means) used to develop the age model for coral M0.4A-3. The chronology is based on linear interpolation between tie-points assigned to mid-April (low Ba/Ca) and mid-October (high Ba/Ca) (see Methods). Black dashed line marks approximate depth to the March 2005 earthquake.

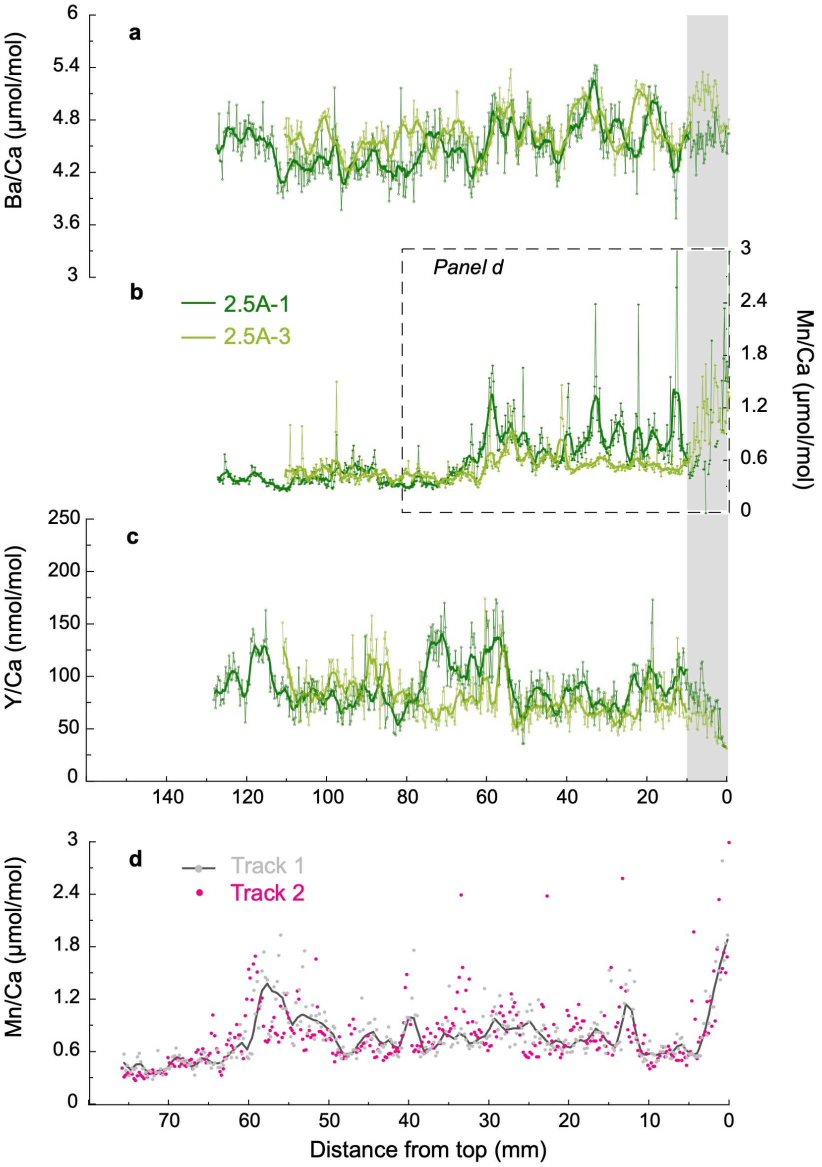

Figure 6 | LA-ICP-MS Ba/Ca, Mn/Ca and Y/Ca data profiles for drill-cores NS09-2.5A-1 and NS09-2.5A-3. (**a-c**) Raw Ba/Ca, Mn/Ca and Y/Ca data (thin lines) and 10-pt running means (thick lines). Grey shading represents the coral tissue layer. (**d**) Replicate analysis of Mn/Ca along two sampling tracks in core 2.5A-1. Mn/Ca data for track 1 were acquired with the major trace element analysis protocol (for Ba, Mn) using a 50 x 500 µm laser spot size. Track 2 Mn/Ca data were acquired with the trace element protocol (for Y) using a 100 x 500 µm laser spot size (following Wyndham et al. 2004). The good replication of the two records shows that the LA-ICP-MS methods are robust and suggests that Mn signals are lattice bound within the coral aragonite.

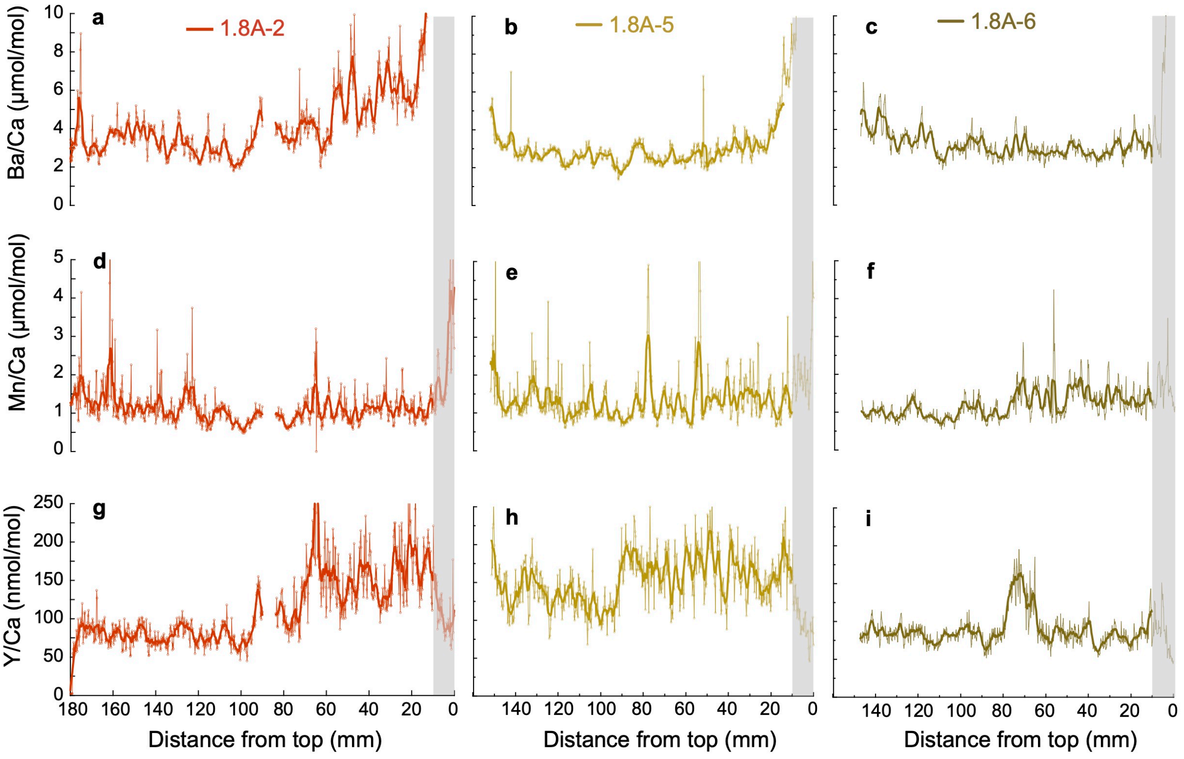

Figure 7 | LA-ICP-MS Ba/Ca, Mn/Ca and Y/Ca data profiles for drill-cores NS09-1.8A-2, NS09-1.8A-5 and NS09-1.8A-6. (**a-c**) Comparison of raw Ba/Ca data (thin lines) and 10-pt running means (thick lines) for the three site 1.8A corals. (**d-f**) As above, but for Mn/Ca. (**g-i**) As above, but for Y/Ca. Grey shading represents the coral tissue layer. The records for coral core 1.8A-2 cover an extended pre-earthquake baseline interval (150–180 mm).

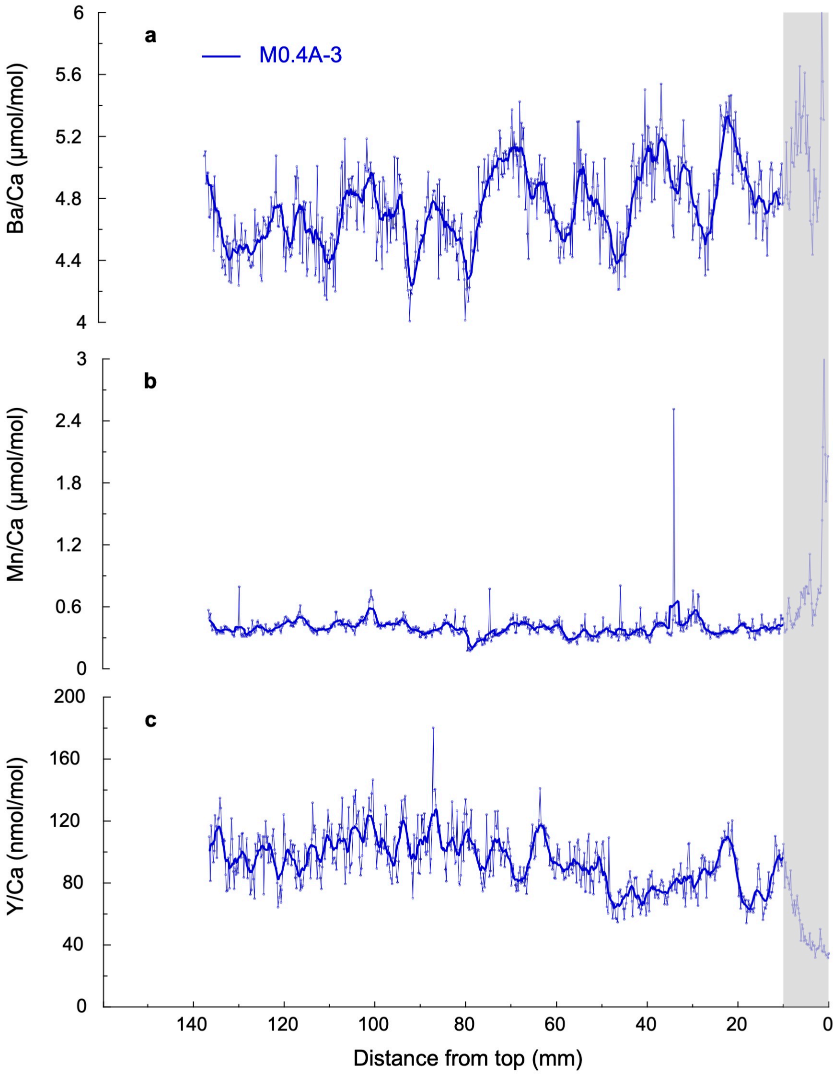

Figure 8 | LA-ICP-MS Ba/Ca, Mn/Ca and Y/Ca data profiles for drill-core NS09-M0.4A-3. (**a-c**) Raw Ba/Ca, Mn/Ca and Y/Ca data (thin lines) and 10-pt running means (thick lines). Grey shading represents the coral tissue layer.

Supplementary Data

Table 1. T-test results for pre- and post-earthquake elemental ratios in the Nias corals. A t-test p-value <0.0001 indicates a significant difference between pre- and post-earthquake mean elemental ratio values. Differences highlighted in blue and orange represent a post-earthquake increase and decrease, respectively. Data within the tissue layer were not included in post-earthquake statistics for Mn/Ca and Ba/Ca.

	Mn/Ca (µmol/mol)							
**Coral ID**	**Pre-EQ Mean**	**Pre-EQ Core SD**	**Pre-EQ Core SE**	**Post-EQ Mean**	**Post-EQ Core SD**	**Post-EQ Core SE**	**ΔEQ**	**T-Test p-value**
2.5A-1	0.39	0.10	0.01	0.82	0.34	0.02	0.43	**<0.0001**
2.5A-3	0.44	0.12	0.01	0.64	0.22	0.01	0.20	**<0.0001**
1.8A-2	0.98	0.37	0.02	1.15	0.39	0.02	0.17	**<0.0001**
1.8A-5	1.24	0.53	0.03	1.42	0.75	0.04	0.18	**<0.0001**
1.8A-6	0.97	0.21	0.01	1.42	0.42	0.02	0.45	**<0.0001**
M0.4A-3	0.40	0.08	0.00	0.41	0.17	0.01	0.01	
	**Y/Ca (nmol/mol)**							
**Coral ID**	**Pre-EQ Mean**	**Pre-EQ Core SD**	**Pre-EQ Core SE**	**Post-EQ Mean**	**Post-EQ Core SD**	**Post-EQ Core SE**	**ΔEQ**	**T-Test p-value**
1.8A-2	84.9	21.4	1.2	158.4	44.4	2.6	73.5	**<0.0001**
1.8A-5	139.2	32.0	1.8	163.8	32.4	1.8	24.6	**<0.0001**
M0.4A-3	102.1	16.7	0.9	81	15.1	0.9	-21.1	**<0.0001**
	**Ba/Ca (µmol/mol)**							
**Coral ID**	**Pre-EQ Mean**	**Pre-EQ Core SD**	**Pre-EQ Core SE**	**Post-EQ Mean**	**Post-EQ Core SD**	**Post-EQ Core SE**	**ΔEQ**	**T-Test p-value**
2.5A-1	4.39	0.25	0.02	4.63	0.29	0.02	0.24	**<0.0001**
2.5A-3	4.56	0.20	0.01	4.74	0.28	0.02	0.18	**<0.0001**
1.8A-2	3.2	0.8	0.04	6.5	2.5	0.17	3.35	**<0.0001**
1.8A-5	2.76	0.67	0.04	2.89	0.67	0.04	0.13	
1.8A-6	3.39	0.78	0.04	2.98	0.51	0.03	-0.41	**<0.0001**
M0.4A-3	4.67	0.26	0.01	4.85	0.28	0.02	0.18	**<0.0001**

Supplementary References

Briggs, R. W. *et al.* Deformation and slip along the Sunda megathrust in the great 2005 Nias-Simeulue earthquake. *Science* **311**, 1897–1901 (2006).

Gagan, M. K. *et al.* Coral $^{13}C/^{12}C$ records of vertical seafloor displacement during megathrust earthquakes west of Sumatra. *Earth Planet. Sci. Lett.* **432**, 461–471 (2015).

Wyndham, T., McCulloch, M. T., Fallon, S. J. & Alibert, C. High-resolution coral records of rare earth elements in coastal seawater: biogeochemical cycling and a new environmental proxy. *Geochim. Cosmochim. Acta* **68**, 2067–2080 (2004).
